# Coffee Versus Caffeine as Ergogenic Aids: Biological and Methodological Distinctions with Implications for Exercise Performance and Recovery

**DOI:** 10.3390/nu18020328

**Published:** 2026-01-20

**Authors:** Przemysław Domaszewski

**Affiliations:** Institute of Health Sciences, University of Opole, Katowicka 68, 45-060 Opole, Poland; przemyslaw.domaszewski@uni.opole.pl

**Keywords:** physical performance, optimization, athletes, sport, safety

## Abstract

*Background*: Caffeine is a well-established ergogenic aid, yet most experimental evidence is based on isolated caffeine, whereas habitual intake in both the general and physically active populations occurs mainly through coffee. This gap between experimental models and everyday practice complicates the interpretation of existing findings. *Objective*: This review compares coffee and isolated caffeine as ergogenic aids, focusing on biological mechanisms, methodological differences, tolerability, and context-dependent use in sport and exercise. *Methods*: A narrative review of human studies examining the effects of coffee and isolated caffeine on exercise performance, fatigue, and post-exercise recovery was conducted, with attention being paid to dosing accuracy, bioavailability, inter-individual variability, and the influence of the coffee matrix. *Results*: Isolated caffeine consistently improves performance under controlled conditions. Coffee can produce similar ergogenic effects, particularly in endurance exercise, although responses are more variable due to differences in caffeine content and individual sensitivity. Emerging evidence suggests that coffee, especially when consumed with carbohydrates, may support post-exercise glycogen resynthesis. Coffee also appears to be better tolerated by many individuals and provides additional bioactive compounds with antioxidant and anti-inflammatory properties. *Conclusions*: Coffee and isolated caffeine should not be viewed as interchangeable ergogenic strategies. While isolated caffeine remains useful in experimental settings, coffee represents a more ecologically relevant and potentially safer source of caffeine in applied practice. Further direct comparative studies are needed to clarify their context-specific roles.

## 1. Introduction

Caffeine is one of the most extensively investigated ergogenic aids in sport and exercise studies [1]. A substantial body of evidence demonstrates that caffeine ingestion can enhance endurance capacity, muscular strength, power output, and aspects of cognitive performance relevant to athletic tasks [2]. As a result, caffeine has been widely incorporated into evidence-based sports nutrition guidelines and position statements [3,4]. Nevertheless, despite the growing body of evidence, most of this literature is based on experiments using isolated caffeine rather than the forms in which caffeine is habitually consumed, which raises important questions about the translational validity of current recommendations.

One notable limitation of the existing caffeine literature is its predominant focus on isolated, anhydrous caffeine, with limited consideration of the conceptual and physiological distinctions between caffeine and coffee. This approach aligns with established experimental methodologies, allowing for repeated dosing with high consistency and precise control over administered caffeine. Accordingly, in laboratory studies caffeine is most commonly provided in capsule or solution form, enabling precise dosing and facilitating clearer interpretation of physiological mechanisms. However, despite its methodological rigor, this experimental model does not reflect typical patterns of caffeine consumption outside the laboratory. Although isolated caffeine supplements are commonly used by athletes, particularly in competitive and experimental settings, habitual caffeine intake in everyday life is predominantly derived from foods and beverages, with coffee representing the primary source among physically active individuals. In practice, many athletes rely mainly on coffee, while others combine coffee consumption with supplemental caffeine depending on training demands, competition schedules, and individual preferences [5].

This discrepancy between experimental models and practical use raises questions regarding the external validity and translational relevance of much of the caffeine literature. Coffee is not merely a source of caffeine but rather a complex food matrix that contains a wide range of bioactive compounds, including, among others, chlorogenic acids, caffeic acid, diterpenes, and various polyphenols [6]. These compounds have been shown to influence glucose metabolism, inflammatory responses, and oxidative stress, suggesting that the physiological effects of coffee consumption may not be fully explained by mechanisms of caffeine alone [7]. Recent evidence further indicates that the biological effects of caffeine are modified by the food and beverage matrix in which it is consumed, including coffee, which can alter absorption, bioavailability, and downstream metabolic responses [8,9]. Consequently, treating coffee and isolated caffeine as functionally equivalent interventions may oversimplify their respective roles in sport and exercise performance and carries the risk of inappropriate extrapolation of findings derived from supplementation studies to habitual dietary coffee consumption.

Evidence examining the ergogenic effects of coffee consumption is comparatively limited and methodologically heterogeneous. Studies vary widely in coffee preparation methods, caffeine content, timing of ingestion, and participant characteristics, contributing to inconsistent findings and greater inter-individual variability in response [10,11]. While several investigations report performance benefits comparable to those observed with isolated caffeine, others demonstrate attenuated or more variable effects [12]. These inconsistencies complicate direct comparisons and have likely contributed to the tendency of guidelines to prioritize caffeine supplementation over coffee consumption [5,13,14].

Another factor contributing to variability in caffeine-related performance outcomes is inter-individual biological differences. Genetic polymorphisms affecting caffeine metabolism, particularly those related to cytochrome P450 1A2 activity, have been proposed as potential modifiers of ergogenic responses [15]. In addition, habitual caffeine intake [16], sex-specific differences [11,17], body composition [18] and individual sensitivity to central nervous system stimulation [19] may influence both the magnitude and direction of performance effects. Recent pharmacokinetic models also show large inter-individual differences in caffeine clearance and peak plasma concentrations that cannot be captured by fixed-dose or body-mass–based dosing alone, further complicating comparisons between isolated caffeine and coffee [20].

These sources of variability are likely to be particularly relevant when caffeine is consumed in the form of coffee, where dosing precision is inherently lower and additional bioactive compounds may further modulate both the pharmacokinetics and pharmacodynamics of caffeine.

Beyond acute performance enhancement, increasing attention has been directed toward the role of nutritional strategies in post-exercise recovery and physiological adaptation [3]. While isolated caffeine has been studied primarily in the context of acute performance, emerging evidence suggests that coffee consumption may influence recovery-related processes, including muscle glycogen resynthesis following endurance exercise [21,22,23]. Such effects are not consistently observed with isolated caffeine and may reflect interactions between caffeine, carbohydrates, and other coffee-derived bioactive compounds. If substantiated, these findings would further differentiate coffee from caffeine as ergogenic strategies with distinct physiological implications.

Despite these considerations, few studies have addressed the differences between coffee and isolated caffeine [24,25,26]. Existing position stands and narrative reviews commonly extend findings from caffeine supplementation studies to coffee consumption without explicitly addressing the methodological and biological limitations of such extrapolation. As a result, practitioners and athletes are often left with simplified recommendations that may not adequately reflect the complexity of caffeine use in real-world settings.

Taken together, these developments in caffeine metabolism, genetic variability, and food matrix science indicate that the assumption of equivalence between coffee and isolated caffeine is no longer tenable and represents a clear gap in the current literature. Therefore, a critical comparison of coffee and isolated caffeine is justified. Such an analysis should not be framed in terms of identifying a universally superior ergogenic aid, but rather in clarifying the contexts in which each approach may be more appropriate. This requires consideration of underlying biological mechanisms, methodological constraints, inter-individual variability, and practical demands related to training, competition, and recovery. The aim of this narrative review is to critically compare coffee and isolated caffeine as ergogenic aids in sport and exercise. Specifically, the review focuses on: (i) the biological mechanisms underlying their ergogenic effects; (ii) methodological challenges associated with interpreting and comparing existing studies; and (iii) context-dependent applications related to performance enhancement and post-exercise recovery. By distinguishing between caffeine supplementation and coffee consumption, this review seeks to provide a more nuanced and physiologically grounded framework for evidence-based decision-making in sports nutrition.

This narrative review was based on a structured search of the scientific literature conducted in PubMed, Scopus, and Web of Science. The main search terms included combinations of caffeine, coffee, exercise, performance, fatigue, recovery, bioavailability, pharmacokinetics, and ergogenic. Additional studies were identified through manual screening of reference lists from relevant reviews and original articles. The review focused on human studies published in peer-reviewed journals that examined the effects of coffee or isolated caffeine on exercise performance, fatigue, or post-exercise recovery. Priority was given to experimental studies, meta-analyses, and recent reviews. Animal studies and papers not directly related to physical performance or recovery were excluded. Because the aim was to provide a critical and mechanistic synthesis rather than a quantitative meta-analysis, formal systematic review procedures (e.g., PRISMA flow diagrams or risk-of-bias scoring) were not applied.

## 2. Methodological Challenges in Comparing Coffee and Isolated Caffeine

Direct comparisons between coffee consumption and isolated caffeine ingestion pose substantial methodological challenges, which complicate both the interpretation of existing evidence and the development of practical recommendations. These challenges stem mainly from differences in dosing precision, intervention standardization, study design, and outcome interpretation. One of the key methodological advantages of isolated caffeine is the ability to administer a precisely controlled dose. In experimental studies, caffeine is typically provided either as an absolute dose (e.g., 200 mg before exercise or 400 mg daily) or as a relative dose expressed per kilogram of body mass. In supplementation research, caffeine is most often administered in capsule or solution form using standardized dose categories, commonly defined as low (~3 mg·kg^−1^), moderate (4–6 mg·kg^−1^), and high (>7 mg·kg^−1^) [27], allowing for reproducibility and clearer dose–response relationships. In contrast, coffee-based interventions inherently involve greater variability in caffeine content, which depends on factors such as coffee species, roast level, grind size, brewing method, interactions between caffeine and other coffee compounds, and serving volume [10]. Even when caffeine content is analytically quantified, substantial variability between servings and across study protocols remains a significant limitation. Moreover, in many studies intake is described in terms of “cups of coffee”, despite evidence that the caffeine content of, i.e., a single espresso serving has been reported to vary widely, ranging from approximately 25 to over 200 mg per serving [28]. Differences in the mode of administration further complicate comparisons. Isolated caffeine is commonly ingested in a fasted or semi-fasted state, whereas coffee is often consumed as part of habitual dietary patterns and may be co-ingested with carbohydrates, milk, or other nutrients. These contextual factors can influence gastric emptying, absorption kinetics, and subjective tolerance, potentially affecting both the magnitude and timing of ergogenic effects. As a result, performance outcomes observed following coffee consumption may reflect not only caffeine exposure but also interactions with the broader dietary matrix, while at the same time more closely mimicking habitual patterns of caffeine intake in real-world settings. To provide a clearer comparative framework, the main methodological differences between isolated caffeine and coffee are summarized in Table 1.

Blinding and placebo control present substantial challenges in studies using coffee as the intervention. Unlike caffeine supplementation, which can be effectively blinded with identical placebo capsules, achieving adequate blinding with coffee is difficult. Although some studies have compared caffeinated and decaffeinated coffee [6,29], such designs do not isolate the effects of caffeine because all other coffee constituents are present in both conditions. As a result, fully placebo-controlled designs are not feasible in this context. These limitations increase the risk of expectancy effects and partial unblinding, which may particularly influence subjective outcomes such as perceived exertion or fatigue.

The heterogeneity of study populations further limits direct comparisons. Supplementation studies often recruit trained athletes or physically active individuals under tightly controlled conditions [27], whereas coffee-based studies frequently involve more heterogeneous samples with varying levels of habitual caffeine intake. Habitual consumption is a particularly important confounder, as tolerance to caffeine’s central nervous system effects may attenuate ergogenic responses [16]. Failure to adequately control or report habitual caffeine intake complicates cross-study comparisons and may partially explain inconsistent findings associated with coffee consumption.

Outcome selection also differs between supplementation and coffee-based research. Isolated caffeine studies predominantly focus on acute performance metrics, such as time to exhaustion, maximal power output, or repetition performance [30,31]. In contrast, coffee-based studies more often include subjective measures, metabolic markers, or recovery-related outcomes [12]. While these outcomes are highly relevant to applied sport contexts, their inclusion introduces additional sources of variability and may limit comparability with traditional ergogenic endpoints.

Finally, few studies have employed head-to-head designs directly comparing coffee and isolated caffeine under equivalent conditions [13,23,25,32]. As a result, conclusions are often derived from indirect comparisons across studies with differing protocols, populations, and outcome measures. This lack of direct comparative evidence necessitates cautious interpretation and underscores the importance of contextualizing findings within their methodological constraints.

An additional limitation affecting the interpretation of both caffeine and coffee studies is publication bias. Studies reporting statistically significant or ergogenic effects are more likely to be published than studies reporting null or negative results, which may lead to an overestimation of the true magnitude and consistency of ergogenic effects in the published literature. This bias is particularly relevant for coffee-based interventions, where methodological heterogeneity and small sample sizes further increase the likelihood that only positive findings reach publication.

Collectively, these methodological challenges highlight that coffee and isolated caffeine cannot be compared solely on the basis of nominal caffeine dose. Differences in intervention delivery, study design, and outcome assessment must be carefully considered when interpreting the ergogenic effects of these two approaches. Addressing these issues is essential for improving the translational relevance of future research and for developing context-specific recommendations for athletes, physically active individuals, as well as non-athletes.

## 3. Ergogenic Effects of Isolated Caffeine Consumption

Isolated caffeine is one of the most consistently supported ergogenic aids in sport and exercise science. Numerous controlled trials and meta-analyses demonstrate that caffeine ingestion enhances endurance performance, muscular strength, power output, and aspects of cognitive function relevant to athletic tasks. These effects have been observed across a wide range of exercise modalities, including continuous endurance exercise, high-intensity intermittent efforts, and resistance training [33,34,35,36]. The primary mechanism underlying the ergogenic effects of caffeine is its antagonistic action on adenosine receptors within the central nervous system [16]. By inhibiting adenosine-mediated signaling, caffeine reduces perceptions of effort and fatigue while increasing alertness and motor drive [37]. This central effect is considered the dominant contributor to performance enhancement, particularly at moderate doses typically ranging from 3 to 6 mg·kg^−1^ body mass [36]. At higher doses, additional peripheral mechanisms, including enhanced calcium release from the sarcoplasmic reticulum, may also contribute to improved muscle contractility [2,38], although the relevance of these mechanisms in humans remains less well established. Evidence supporting caffeine’s ergogenic effects is particularly robust in endurance-based activities. Multiple studies report increased time to exhaustion, improved time-trial performance, and reduced ratings of perceived exertion following caffeine ingestion [19,39,40]. In resistance and power-based exercise, caffeine has been shown to increase repetition performance, peak power, and maximal voluntary force, although the magnitude of these effects appears more variable and may depend on training status and habitual caffeine use [1,14,41,42,43].

Despite the overall consistency of findings, substantial inter-individual variability in responses to isolated caffeine is well documented. Genetic polymorphisms influencing caffeine metabolism, particularly within the *CYP1A2* gene, have been proposed as important modifiers of ergogenic responses; however, evidence remains inconsistent across exercise modalities and study designs. In many studies, inclusion criteria explicitly exclude individuals with caffeine hypersensitivity [18,41], which may indirectly limit the participation of individuals carrying certain *CYP1A2* polymorphisms. Habitual caffeine intake further complicates interpretation, as tolerance to caffeine’s central effects may reduce performance benefits in regular consumers [16,33,44]. Consequently, although isolated caffeine allows a high level of dosing precision and reproducibility, its ergogenic effects are not uniform across individuals.

From a methodological perspective, isolated caffeine provides strong internal validity through precise dosing, effective blinding, and standardized protocols. However, these advantages also limit ecological validity, as isolated caffeine does not reflect typical dietary intake. Overall, isolated caffeine remains a reliable ergogenic aid under controlled conditions and a useful benchmark for evaluating other caffeine-containing strategies, including coffee, although its applicability beyond experimental settings should be interpreted cautiously.

## 4. Ergogenic Effects of Coffee Consumption

Coffee consumption has been investigated as an ergogenic strategy in a smaller and more heterogeneous body of studies than isolated caffeine. Despite this, the available evidence indicates that coffee can improve exercise performance, particularly during endurance exercise [25,45,46,47]. In many of these studies, improvements in endurance performance, time to exhaustion, and ratings of perceived exertion are similar to those reported for isolated caffeine when the estimated caffeine dose provided by coffee is comparable [25]. At the same time, the size of the effect and its consistency tend to be more variable than in studies using standardized caffeine supplementation.

Caffeine content alone does not fully account for all reported findings. Some studies have shown performance improvements after the ingestion of decaffeinated coffee, which points to the possible involvement of factors other than caffeine itself. These may include expectancy and placebo-related effects, as well as the contribution of other bioactive compounds naturally present in coffee [6,48]. In addition, coffee-based interventions differ widely in caffeine content, brewing methods, and timing of ingestion. This lack of standardization complicates comparisons across studies and likely contributes to greater interindividual variability in performance responses than is typically observed with isolated caffeine.

Although the ergogenic effects of coffee are usually attributed mainly to its caffeine content and its action on adenosine receptors [49], coffee is not simply a source of caffeine. It is a complex food matrix that also contains chlorogenic acids, caffeic acid, diterpenes, and other polyphenols. These compounds have been linked to changes in glucose metabolism, insulin sensitivity, and inflammatory responses, suggesting that the physiological effects of coffee may extend beyond those of caffeine alone [50,51,52].

Mechanistically, several of these compounds can modulate both the pharmacokinetics and the physiological actions of caffeine. Chlorogenic acids and related polyphenols may slow gastric emptying and influence intestinal transport processes, leading to a more gradual rise in plasma caffeine concentration compared with isolated caffeine. This can attenuate acute overstimulation while prolonging central nervous system exposure. In parallel, some coffee constituents interact with hepatic cytochrome P450 enzymes, including CYP1A2, which is responsible for most caffeine metabolism, potentially altering the rate of caffeine clearance and the formation of active metabolites such as paraxanthine. Beyond caffeine metabolism, coffee polyphenols and melanoidins exert antioxidant, anti-inflammatory, and vasomodulatory effects that may interact with caffeine’s ergogenic mechanisms. For example, improvements in endothelial function and nitric oxide–mediated blood flow could enhance oxygen and substrate delivery to working muscle, while reductions in oxidative and inflammatory stress may influence fatigue development during prolonged exercise.

Evidence for ergogenic effects of coffee in resistance exercise and high-intensity tasks is less consistent. Some studies report improvements in muscular endurance, power output, or jumping performance after coffee ingestion, whereas others show little or no benefit [23,53]. Differences in training status, habitual caffeine intake, task specificity, and study design likely contribute to these mixed findings. Compared with isolated caffeine, coffee ingestion may also produce a lower and more prolonged plasma caffeine peak, which could reduce its effectiveness in activities requiring maximal or rapid neuromuscular activation [54].

Interest has also grown in the potential role of coffee in post-exercise recovery. Several studies suggest that coffee intake, especially when combined with carbohydrates, may enhance muscle glycogen resynthesis during recovery after endurance exercise [21]. Such effects are not consistently observed with isolated caffeine and may reflect interactions between caffeine, carbohydrate availability, and other coffee-derived compounds. However, the number of studies addressing recovery outcomes remains limited, and the available evidence should be interpreted with caution.

From a practical standpoint, coffee has clear advantages. It is widely available, familiar to most athletes, and easily incorporated into daily routines. At the same time, variability in caffeine content, gastrointestinal tolerance, and expectancy-related effects are important limitations, particularly in competitive settings where precise and reproducible dosing is required.

Overall, coffee can produce ergogenic effects that broadly resemble those of isolated caffeine across many exercise contexts. However, greater variability in responses, methodological differences between studies, and the influence of the coffee matrix distinguish coffee consumption from caffeine supplementation. The presence of additional bioactive compounds in coffee may contribute to metabolic, oxidative, and inflammatory processes relevant to both performance and recovery. For these reasons, coffee is best viewed not as a simple substitute for isolated caffeine, but as a distinct, context-dependent ergogenic strategy.

## 5. Bioavailability, Pharmacokinetics and Inter-Individual Variability

The ergogenic effects of both isolated caffeine and coffee consumption are shaped to a large extent by differences in bioavailability, pharmacokinetics, and inter-individual variability [55]. Although caffeine is the primary compound responsible for performance enhancement, its absorption, metabolism, and physiological effects depend not only on the ingested dose but also on the form in which it is consumed and on individual biological characteristics [56].

Caffeine is rapidly absorbed from the gastrointestinal tract, with peak plasma concentrations typically observed between 30 and 120 min after ingestion [57]. More recent studies suggest that optimal ergogenic effects may occur closer to ~30 min post-ingestion rather than the traditionally recommended 40–60 min proposed in earlier guidelines [1]. Oral bioavailability is close to 100%, and absorption is generally not constrained by age or sex [12]. However, caffeine metabolism may differ between sexes, which has been partly attributed to differences in body composition, including body fat content [11,16,18,27]. However, the time course of caffeine appearance in the bloodstream differs between isolated caffeine and coffee [24,47,58]. When caffeine is consumed as coffee, peak plasma concentrations tend to be slightly delayed and more variable than after capsule ingestion. This is likely related to differences in gastric emptying, beverage volume, and interactions with other coffee constituents [54]. Such kinetic variability provides a plausible explanation for the less consistent performance responses observed after coffee ingestion.

Following absorption, caffeine is metabolized primarily in the liver by cytochrome P450 1A2 (CYP1A2), which accounts for approximately 95% of caffeine clearance [54,59]. Genetic polymorphisms in the *CYP1A2* gene have therefore been proposed as modifiers of caffeine metabolism rate, with individuals carrying the AA genotype generally classified as faster metabolizers than AC or CC carriers [15,59]. Some studies suggest that slower metabolism may prolong ergogenic effects, but results remain inconsistent across exercise modalities and populations [13,36]. Overall, genotype effects appear to be context-dependent and should not be interpreted as deterministic predictors of performance outcomes.

Beyond genetic variation, habitual caffeine intake represents another important source of inter-individual variability. Regular caffeine consumers may develop partial tolerance to its central and perceptual effects, potentially reducing changes in perceived exertion or alertness after acute ingestion [46]. This issue is particularly relevant in coffee-based studies, as habitual coffee consumption is common among physically active individuals and is not always adequately controlled or reported. Inconsistent assessment of habitual intake complicates cross-study comparisons and likely contributes to heterogeneous findings [60,61].

Sex-specific factors represent an additional, yet still underexplored, source of variability. Hormonal fluctuations, oral contraceptive use, and sex-related differences in caffeine clearance may all influence caffeine pharmacokinetics and responsiveness in women [11,18,62]. Ovarian hormones modulate hepatic CYP1A2 activity, leading to phase-dependent differences in caffeine clearance across the menstrual cycle [17]. During the luteal phase, when progesterone and estrogen levels are elevated, caffeine metabolism is generally slower, resulting in higher and more prolonged plasma caffeine concentrations than during the follicular phase. The use of estrogen-containing oral contraceptives further suppresses CYP1A2 activity and can reduce caffeine clearance by more than 50%, effectively doubling caffeine half-life in some women [54]. Consequently, women using hormonal contraception may experience stronger and longer-lasting central nervous system and cardiovascular responses to the same nominal caffeine dose, with implications for both ergogenic effects and the risk of adverse reactions.

Despite this, women remain underrepresented in both caffeine supplementation and coffee-based performance studies, which limits the generalizability of current evidence [13,63]. Failure to control for menstrual cycle phase and contraceptive use therefore represents a major, often unacknowledged source of variability in caffeine research involving women.

Taken together, these observations indicate that variability in bioavailability, metabolism, and individual sensitivity substantially shapes the ergogenic effects of both isolated caffeine and coffee. As a result, identical nominal caffeine doses can lead to different physiological and performance outcomes depending on the caffeine source and individual characteristics. This complexity reinforces the need for context-aware interpretation of caffeine research and cautions against assuming equivalence between coffee consumption and isolated caffeine supplementation in applied sport settings.

## 6. Coffee Versus Caffeine in Post-Exercise Recovery

While the ergogenic effects of both coffee and isolated caffeine have been extensively examined in the context of acute exercise performance, considerably less attention has been paid to their respective roles in post-exercise recovery. Although coffee and isolated caffeine share caffeine as a common active component, they differ substantially in biological complexity, which may have implications for recovery-related processes.

In the recovery context, isolated caffeine appears to influence post-exercise processes primarily through mechanisms related to metabolic regulation and substrate availability. Caffeine has been reported to affect glucose metabolism and, when co-ingested with carbohydrates, may enhance post-exercise muscle glycogen resynthesis. However, caffeine exhibits context-dependent, dual pro- and anti-inflammatory responses, with the magnitude and direction of these effects influenced by dose, timing, and metabolic state, potentially contributing to variability in inflammatory signaling and tissue recovery following exercise [12,24,60]. Mechanistically, increases in glycogen resynthesis observed following caffeine ingestion have been primarily attributed to elevated insulin concentrations and enhanced glucose transport into skeletal muscle and the liver [22,25,64]. Additional mechanisms, such as suppression of glycogen phosphorylation and the potential involvement of calcium/calmodulin-dependent protein kinase-related signaling pathways, have also been proposed; however, these effects appear to be strongly dependent on high carbohydrate availability and adequate glucose intake [22]. Taken together, current evidence suggests that caffeine per se is unlikely to act as a primary driver of post-exercise glycogen resynthesis, but may facilitate recovery under specific nutritional conditions.

In contrast to isolated caffeine, coffee represents a complex dietary source of caffeine embedded within a matrix of additional bioactive compounds. Beyond caffeine itself, coffee contains polyphenols, diterpenes, melanoidins, and other constituents that may modulate recovery-related processes through multiple, partly overlapping pathways. These include modulation of inflammatory and oxidative stress responses [65], influences on glucose metabolism [21], effects on vascular function via nitric oxide-related pathways [66], and interactions with the gut microbiota [67,68,69]. Randomized trials in endurance-trained individuals have demonstrated that post-exercise ingestion of coffee-containing carbohydrate beverages results in greater muscle glycogen accumulation during recovery compared with carbohydrate intake alone [21]. Similarly, studies employing high doses of isolated caffeine co-ingested with carbohydrates have reported enhanced glycogen resynthesis relative to carbohydrate intake alone [22]. Notably, the magnitude of glycogen resynthesis observed in coffee-based interventions appears greater than that typically reported in studies using isolated caffeine, suggesting that components of the coffee matrix beyond caffeine itself may contribute to this response. However, the absence of direct, head-to-head comparisons between coffee and isolated caffeine under matched conditions precludes definitive conclusions.

The observed effects of coffee on glycogen restoration appear to be most pronounced during the early phases of recovery, when skeletal muscle exhibits heightened insulin-independent glucose uptake. The mechanisms underlying these observations are likely multifactorial. Coffee may influence glycogen storage through caffeine-related mechanisms while simultaneously providing additional bioactive compounds, such as caffeic acid and cafestol, which have been shown to influence glucose metabolism, insulin secretion, and intracellular signaling pathways associated with glycogen synthesis and glucose uptake in skeletal muscle [26,51]. Coffee-derived compounds may further influence recovery through interactions with the gut microbiota. Chlorogenic acids (CGAs), which constitute the major polyphenolic fraction of coffee, are only partially absorbed in the small intestine and are extensively metabolized by colonic bacteria into smaller phenolic acids, including caffeic and ferulic acid derivatives, which are subsequently reabsorbed and considered to represent the biologically active forms responsible for many systemic effects attributed to CGAs [69,70]. In addition, melanoidins formed during coffee roasting act as dietary fiber–like compounds that reach the colon largely intact, where they serve as substrates for microbial fermentation and promote the release of phenolic compounds with antioxidant activity and increased bioaccessibility [71]. Through these combined mechanisms, habitual coffee consumption has been associated with alterations in gut microbiota composition, including increases in bacterial taxa linked to improved glucose metabolism and insulin sensitivity [67,72]. In parallel, coffee polyphenols have been shown to influence vascular function via nitric oxide-related pathways, potentially facilitating nutrient delivery and supporting metabolic recovery processes [73,74,75].

The combined presence of caffeine and other coffee-derived compounds may therefore create a metabolic environment more favorable to glycogen restoration than isolated caffeine alone. Importantly, these recovery-related effects appear to be highly context-dependent. Beneficial effects of coffee on glycogen resynthesis have primarily been observed following prolonged endurance exercise resulting in substantial glycogen depletion and when coffee is consumed alongside adequate carbohydrate doses [25]. Whether similar effects occur following resistance exercise, high-intensity intermittent activity, or in the absence of carbohydrate co-ingestion remains unclear. Moreover, the limited number of available studies, relatively small sample sizes, and substantial methodological heterogeneity warrant cautious interpretation. The interpretation of these findings is further constrained by important methodological limitations in the existing recovery literature. Most studies investigating coffee- or caffeine-enhanced glycogen resynthesis involve small sample sizes, often fewer than 10–15 participants per condition, which limits statistical power and increases the risk of inflated effect estimates. In addition, pre-exercise glycogen status, habitual caffeine intake, and macronutrient consumption in the days preceding testing are not always standardized or adequately reported, despite their strong influence on post-exercise recovery. Training status and prior exercise load are also inconsistently controlled, further complicating the attribution of observed effects specifically to coffee or caffeine. As a result, current evidence should be considered preliminary rather than definitive.

From an applied perspective, enhanced glycogen resynthesis may be particularly relevant for athletes facing short recovery intervals between training sessions or competitive events [76,77]. In such scenarios, coffee consumption may offer practical advantages over isolated caffeine by simultaneously providing the well-established ergogenic effects of caffeine and additional bioactive compounds that may support metabolic and inflammatory aspects of recovery. However, potential trade-offs, including gastrointestinal tolerance must be considered.

In summary, current evidence suggests both convergence and divergence between coffee and isolated caffeine with respect to post-exercise recovery. While isolated caffeine primarily acts as an acute ergogenic aid with limited recovery-specific effects, coffee consumption may additionally support recovery-related processes, particularly muscle glycogen resynthesis, under specific nutritional and exercise conditions. These observations support the view that coffee and isolated caffeine should be considered related but distinct, context-dependent strategies rather than interchangeable interventions.

## 7. Tolerability, Adverse Effects and Practical Constraints

Both isolated caffeine and coffee are generally well tolerated when consumed within commonly recommended doses; however, their tolerability profiles and practical limitations differ in ways that are relevant in applied sport settings. Adverse effects associated with caffeine intake may include gastrointestinal discomfort, anxiety, elevated heart rate, and sleep disturbances, although the occurrence and severity of these symptoms vary markedly between individuals [18,36,78].

Isolated caffeine supplementation, particularly in anhydrous form, appears to be associated with a higher risk of acute side effects, especially when consumed at moderate to high doses (≥6 mg·kg^−1^ body mass). Rapid absorption and higher peak plasma caffeine concentrations may intensify symptoms such as nervousness, palpitations, or gastrointestinal distress in susceptible individuals. In some sports, particularly those requiring fine motor control or sustained concentration, such effects may negatively affect performance rather than enhance it [54].

For some individuals, coffee may be better tolerated. A slower rise in plasma caffeine concentration, together with the presence of other coffee constituents that may influence gastric emptying and central nervous system stimulation, could partly explain these differences [65,79]. However, coffee is not universally well tolerated. Its acidity, temperature, and volume can provoke gastrointestinal symptoms, especially when consumed shortly before high-intensity exercise [23]. In addition, individual sensitivity to coffee’s sensory characteristics may shape expectancy effects and subjective responses [23].

Sleep disruption is a key practical concern for both sources of caffeine. Consumption later in the day has been shown to impair sleep quality and reduce total sleep time, with potential negative consequences for recovery and subsequent performance [6,80]. These effects may be more pronounced in slower caffeine metabolizers or in individuals with high habitual caffeine intake who continue to consume caffeine across the day [54].

From a practical perspective, isolated caffeine offers clear advantages in terms of dosing precision, portability, and reproducibility, which can be important in elite or tightly controlled competition settings. Coffee, in contrast, is easily incorporated into daily routines and training environments but presents challenges related to standardization, timing of intake, and individual tolerance.

## 8. When Coffee, When Caffeine?

Given the differences in bioavailability, physiological effects, tolerability, and practical constraints, coffee and isolated caffeine should be viewed as context-dependent ergogenic strategies rather than interchangeable interventions. The selection of an appropriate caffeine source depends on the specific demands of training, competition, and recovery. In competitive settings requiring precise timing and reproducible ergogenic effects, such as elite endurance events or standardized performance tests, isolated caffeine supplementation may be preferable due to its controlled dosing and predictable pharmacokinetics. This approach allows athletes to target specific plasma caffeine concentrations and minimize variability in response.

In contrast, coffee consumption may be better suited to training environments, longer-duration sessions, or scenarios where ecological validity and habitual dietary practices are prioritized. For recreational athletes and habitual coffee consumers, coffee provides a familiar and accessible means of caffeine ingestion that may enhance performance while maintaining comfort and compliance. The slower and more variable absorption profile of coffee may also reduce the likelihood of acute overstimulation in sensitive individuals.

The recovery context represents another domain in which coffee and isolated caffeine may differ in applicability. Emerging evidence suggests that coffee consumption, particularly when combined with carbohydrate intake, may support post-exercise muscle glycogen resynthesis following endurance exercise [21,72]. In these studies, coffee was typically co-ingested with carbohydrates, a pattern that also reflects habitual consumption practices in many individuals who regularly add sugar to coffee. Such habitual behaviors may inadvertently create nutritional conditions favorable for glycogen restoration during recovery. In this context, coffee may offer benefits extending beyond acute stimulation, while isolated caffeine has also been shown to facilitate glycogen resynthesis when co-ingested with carbohydrates, although its effects appear more dependent on dosing and nutritional context.

Temporal factors also shape practical decision-making. Caffeine consumed earlier in the day is less likely to interfere with subsequent sleep, whereas intake later in the day may carry a higher risk of residual stimulation, particularly in individuals with greater sensitivity or slower caffeine clearance. For this reason, personal tolerance, habitual intake, and prior experience remain important considerations when selecting and timing caffeine- or coffee-based strategies. Overall, the available evidence does not justify a general recommendation in favor of either coffee or isolated caffeine. Rather, their optimal use appears to depend on the specific performance or recovery goal, the methodological context, and individual characteristics. Acknowledging these context-dependent differences is important for translating findings from caffeine research into practical and workable strategies in sport nutrition.

## 9. Limitations and Future Research Directions

Despite substantial evidence supporting the ergogenic effects of caffeine, several limitations constrain comparisons between coffee and isolated caffeine. Most importantly, well-controlled, head-to-head studies directly comparing these two sources under equivalent conditions remain scarce. As a result, current conclusions are largely based on indirect evidence. Coffee-based interventions also present methodological challenges due to variability in preparation methods, caffeine content, and co-ingestion with other nutrients, which limits standardization and comparability across studies. In addition, inter-individual factors such as habitual caffeine intake, sex-related differences, and variability in caffeine metabolism are not consistently controlled, particularly in studies involving habitual coffee consumers. Another key limitation is the focus on acute outcomes. Long-term effects of repeated coffee or caffeine use on recovery and training adaptation remain poorly understood, and evidence related to post-exercise recovery is based on a small number of studies with limited sample sizes.

The primary aim of this review is therefore to draw attention to the widespread use of coffee; the most commonly consumed psychoactive substance worldwide; as a source of caffeine, and to highlight the need for experimental studies that directly test differences between coffee and isolated caffeine. Future research should prioritize controlled, comparative designs to clarify whether and when these sources can be considered equivalent in applied sport contexts.

## 10. Conclusions

In applied settings, caffeine intake is dominated by coffee consumption, making coffee the most ecologically relevant source of caffeine among physically active individuals. Coffee provides ergogenic effects broadly comparable to those of isolated caffeine, particularly in endurance exercise, while responses tend to be more variable due to differences in preparation, caffeine content, and individual sensitivity. Importantly, coffee appears to be better tolerated by many individuals, with a lower incidence of acute adverse effects compared with isolated caffeine, especially at moderate doses. This more favorable tolerability profile, together with the presence of additional bioactive compounds with antioxidant and anti-inflammatory properties, suggests that coffee may offer a safer and more protective means of caffeine delivery. Thus, while coffee and isolated caffeine share a common ergogenic mechanism, the broader bioactive matrix of coffee may confer advantages in terms of safety and overall physiological impact, supporting the view that these sources should be considered related but distinct, context-dependent strategies rather than interchangeable interventions.

## Figures and Tables

**Table 1:** Key methodological differences between isolated caffeine and coffee as ergogenic interventions.

Dimension	Isolated Caffeine	Coffee
Dose control	Precisely quantified (mg or mg·kg^−1^)	Highly variable; depends on coffee type, preparation and serving size
Standardization	High; capsules and solutions allow reproducible protocols	Low; preparation and caffeine content vary widely
Blinding	Strong; placebo capsules easily matched	Limited; caffeinated vs. decaffeinated coffee does not control for non-caffeine compounds
Bioavailability	Rapid and predictable absorption	Slower and more variable due to beverage matrix
Inter-individual variability	Driven mainly by genetics and habitual intake	Amplified by matrix effects and dose imprecision
Ecological validity	Low; rarely reflects habitual intake	High; reflects real-world caffeine consumption
Expectancy effects	Relatively low	Higher due to sensory cues and beliefs about coffee
Recovery relevance	Usually not assessed	Often co-ingested with meals or carbohydrates
Risk of bias	Publication bias towards positive findings	Publication bias plus greater within-study methodological variability

## Data Availability

No new data were created or analyzed in this study.

## Abbreviations

The following abbreviations are used in this manuscript:

AA	homozygous CYP1A2 A allele
AC	heterozygous CYP1A2 genotype
CC	homozygous CYP1A2 C allele
CGA	chlorogenic acids
CNS	central nervous system
CYP1A2	cytochrome P450 1A2
mg·kg^−1^	milligrams per kilogram of body mass
NO	nitric oxide
PK	pharmacokinetics
RPE	rating of perceived exertion
Tmax	time to peak plasma concentration
